# Pediatric Granulomatosis With Polyangiitis: A Case Report Compared to a Case Review in the Last 10 Years

**DOI:** 10.7759/cureus.77239

**Published:** 2025-01-10

**Authors:** Mariana Costin, Eliza Cinteza, Anca Croitoru, Irina Popescu, Marcela Daniela Ionescu

**Affiliations:** 1 Pediatric Nephrology, Emergency Clinical Hospital for Children "Maria Sklodowska Curie", Bucharest, ROU; 2 Pediatric Cardiology, Emergency Clinical Hospital for Children "Maria Sklodowska Curie", Bucharest, ROU; 3 Pediatric Radiology, Emergency Clinical Hospital for Children "Maria Sklodowska Curie", Bucharest, ROU; 4 Pediatrics, Emergency Clinical Hospital for Children "Maria Sklodowska Curie", Bucharest, ROU

**Keywords:** c-anca vasculitis, children, end-stage kidney disease, glomerulonephritis, granulomatosis with polyangiitis (gpa), proteinuria

## Abstract

Granulomatosis with polyangiitis (GPA), previously described as Wegener’s granulomatosis, is a rare autoimmune disease that causes necrotizing systemic vasculitis, affecting small- and medium-sized blood vessels. While adults typically receive GPA diagnoses more frequently, children and adolescents can also experience it, albeit infrequently. In pediatric patients, the disease presents challenges due to its rarity and complex clinical symptoms. Early detection and treatment are essential for minimizing long-term complications and improving outcomes. We conducted a literature review that included cases of GPA in children reported between 2014 and 2024, focusing on their unique clinical presentation and outcomes. In this report, we present a seven-year-old girl characterized by an atypical clinical course that included neurological manifestations, particularly posterior reversible encephalopathy syndrome (PRES) and rapidly progressive glomerulonephritis, resulting in end-stage kidney disease, which required hemodialysis. After analyzing all the data, we found that the patient had been diagnosed with GPA. Clinical symptoms are similar to those reported in the literature. These symptoms include female sex and constitutional symptoms (fever, weight loss, fatigue), arthralgia, and multiple organ involvement (hemoptysis, an image demonstrating pulmonary nodules and peribronchovascular infiltrates), as well as otorhinolaryngological symptoms (including nasal crusting and rhinitis history), along with renal impairment (proteinuria, hematuria, and glomerulonephritis pattern). Our case differs from others in the literature due to its early onset (the average age at diagnosis in the recent research was 12 years old), the patient’s poor response to cyclophosphamide (CYC) treatment (she developed right lower and middle lobe pneumonia and hemoptysis), and the unexpected changes in her symptoms. She experienced several paroxysmal events, including confusion, blurred vision, generalized seizures with tonic-clonic movements, and deviation of her eyes to the right side, which was followed by temporary aphasia. These neurological symptoms prompted us to conduct a more thorough investigation, leading us to identify a homozygote mutation C667T in the methylenetetrahydrofolate reductase gene (MTHFR). This case highlights the importance of a high index of suspicion for GPS in patients with constitutional symptoms (fever, weight loss, fatigue), arthralgia, and multiple organ involvement, as well as early detection and timely treatment.

## Introduction

Granulomatosis with polyangiitis (GPA) is an autoimmune disorder characterized by necrotizing systemic vasculitis that primarily affects small- and medium-sized blood vessels, including small arteries, arterioles, venules, and capillaries, with involvement of the kidneys and the respiratory system. This condition typically presents with symptoms such as chronic sinusitis, systemic manifestations, recurrent pneumonia, glomerulonephritis, and various skin lesions (including papules, vesicles, and ulcers) [1]. The diagnosis of GPA can be made if at least three of the six following EULAR/PReS/PRINTO criteria are met: 1) histopathology (granulomatous inflammation within the arterial wall or in the perivascular or extravascular area); 2) upper airway involvement (chronic purulent or bloody nasal discharge or recurrent epistaxis/crusts/granulomata, nasal septum perforation or saddle nose deformity, chronic or recurrent sinus inflammation); 3) laryngo-trachea-bronchial stenoses (subglottic, tracheal, or bronchial stenosis); 4) pulmonary involvement (chest X-ray or CT scan showing the presence of nodules, cavities or fixed infiltrates); 5) anti-neutrophil cytoplasm antibodies (ANCA) positivity by immunofluorescence or ELISA (P-ANCA/MPO-ANCA or C-ANCA/PR3-ANCA); 6) and renal involvement (proteinuria > 0.3 g/24 h or > 30 mmol/mg of urine albumin-creatinine ratio on a spot morning sample, hematuria or red blood cell casts in the urinary sediment or ≥2+ on dipstick, or necrotizing pauci-immune glomerulonephritis) [2].

While GPA is predominantly diagnosed in adults, it can also manifest in children and adolescents, but with far lower frequency. The disease predominantly impacts persons in their fourth to sixth decades of life, with the median age of the pediatric onset being 12-14 years old. The incidence rate of GPA in the pediatric population is 1.8 per 1,000,000 in contrast to 12 per 1,000,000 in adults. Childhood-onset GPA, characterized by onset prior to age 18, exhibits numerous similarities with adult-onset GPA, including a female predominance (with a male-to-female ratio of 1:2 in children), yet also presents several notable distinctions. It predominantly affects the ear, nose, and throat (ENT) regions and is linked to systemic symptoms, including renal, lower respiratory tract, musculoskeletal, and skin involvement. Moreover, childhood-onset GPA is associated with an elevated risk of serious complications. This condition is associated with considerable long-term morbidity, marked by frequent relapses. EULAR/ERA-EDTA (European League Against Rheumatism/ European Renal Association-European Dialysis and Transplant Association) and CanVasc recommend the use of a combination of glucocorticoids and either CYC or rituximab (RTX) as the primary approach for remission induction therapy. The long-term prognosis for children with GPA depends on the severity of renal involvement, as early kidney loss may result in complications such as the necessity for dialysis or renal transplantation. Consequently, prompt diagnosis and intervention are essential for preventing complications and improving outcomes.

## Case presentation

We present the case of a seven-year-old girl who was referred to our clinic with a diagnosis of acute kidney injury. The patient was initially admitted to a regional hospital for malaise, nausea, abdominal pain, myalgia, and a persistent low-grade fever that began 10 days earlier.

Throughout her two-day admission to the regional hospital, the patient's condition worsened, evidenced by the onset of hematuria with proteinuria, a rapid increase in serum creatinine, a notable inflammatory response, and moderate hepatic cytolysis, which raised concerns for acute glomerulonephritis. As a result, she was referred to our pediatric nephrology department for additional management.

At the time of admission in our department, the patient exhibited mottled skin on the extremities, pallor, malaise, headache, myalgia, and a productive cough accompanied by vomiting and hemoptysis. The patient reported pain in the coxofemoral joint, along with hyperemia of the overlying tissue and pain in the proximal interphalangeal joint of the right hand's second finger.

Reviewing her history indicated that the patient had experienced migratory arthralgia for the past three months, marked by arthritic pain without joint swelling or functional limitation, with temporary relief following treatment with a third-generation cephalosporin for presumed ‘infectious arthritis’. She also had a two-month history of recurrent mild episodes of epistaxis that resolved spontaneously.

She possesses no notable personal medical history and has attained all anticipated developmental milestones, and her vaccinations are current. There is no history of renal or autoimmune disorders in the family.

Laboratory studies revealed microcytic hypochromic anemia, mild leukocytosis with neutrophilia, minor eosinophilia, and markedly elevated inflammatory markers. The evaluation of renal function revealed increased serum urea, creatinine, and uric acid levels, indicating compromised renal function. The liver enzymes, including alanine aminotransferase (ALT), aspartate aminotransferase (ALP), and gamma-glutamyl transferase (GGT), were high, signifying hepatic dysfunction. Furthermore, her serum albumin level was low at 3.32 g/dL, and the prothrombin time (PT) was extended to 15.9 seconds. The ionogram indicated mild hyponatremia along with hyperkalemia (Table 1). 

**Table 1:** Initial laboratory results of the patient at presentation Hb = hemoglobin, MCV = mean corpuscular volume, MCH = mean corpuscular hemoglobin, ESR = erythrocyte sedimentation rate, LT = alanin aminotransferase, GGT = gamma-glutamyl transferase, AST = aspartate aminotransferase, PT = prothrombin time, Ig G = immunoglobulin G

Laboratory parameter	Patient’s values	Normal values
Hb	8.9 g/dL	11.5-14.5 g/dL
MCV	69.5 fL	75.0-89.0 fL
MCH	22.1 pg	25.0-31.0 pg
CRP	302.4 mg/L	0-5.0 mg/L
Procalcitonin	3.78 ng/mL	< 0.05 ng/mL
ESR	120 mm/h	2.0-25.0 mm/h
Fibrinogen	702 mg/dL	140.0-360.0 mg/dL
Ferritin	1633 ng/mL	7.0-84.0 ng/mL
Serum creatinine	3.38 mg/dL	0.3-0.7 mg/dL
Serum urea	57.2 mg/dL	5-20 mg/dL
ALT	82.4 U/L	2.0-37.0 U/L
AST	56.1 U/L	2.0-44.0 U/L
GGT	94 U/L	3.0-31.0 U/L
Serum albumin	3.32 g/dL	3.8-5.4 g/dL
PT	15.9 seconds	11.3-15.6 seconds
Serum sodium	137.2 mmol/L	138.0-145.0 mmol/L
Serum potassium	5.38 mmol/L	3.5-5.1 mmol/L
Ig G	1650.6 mg/dL	572.0-1474.0 mg/dL

We evaluated potential infectious causes, systemic vasculitis, and autoimmune diseases for the differential diagnosis. Screening for infectious diseases revealed negative results for hepatitis C antibodies (HCV), hepatitis B surface antigen (HBsAg), HIV 1/2 antibodies, and IgG/IgM antibodies against SARS-CoV-2. Blood cultures were sterile, ruling out infectious etiologies of acute glomerulonephritis.

The immunological profile, which includes anti-streptolysin (ASLO), C3, C4, total hemolytic complement (CH50), and an extended antinuclear antibody (ANA) profile, yielded normal results, thereby excluding autoimmune conditions such as systemic lupus erythematosus and Sjögren syndrome. The tests for anti-glomerular basement membrane (anti-GBM) antibodies, perinuclear ANCA (p-ANCA) antibodies, and anticardiolipin antibodies returned negative results, therefore excluding Goodpasture syndrome, p-ANCA-associated vasculitis (e.g., eosinophilic granulomatosis with polyangiitis and microscopic polyangiitis), and antiphospholipid syndrome. The anti-neutrophil cytoplasmic (c-ANCA) antibodies were positive (213.666 UI/mL), surpassing normal values by over tenfold, strongly suggesting granulomatosis with polyangiitis as the primary diagnosis.

**Figure 1:**
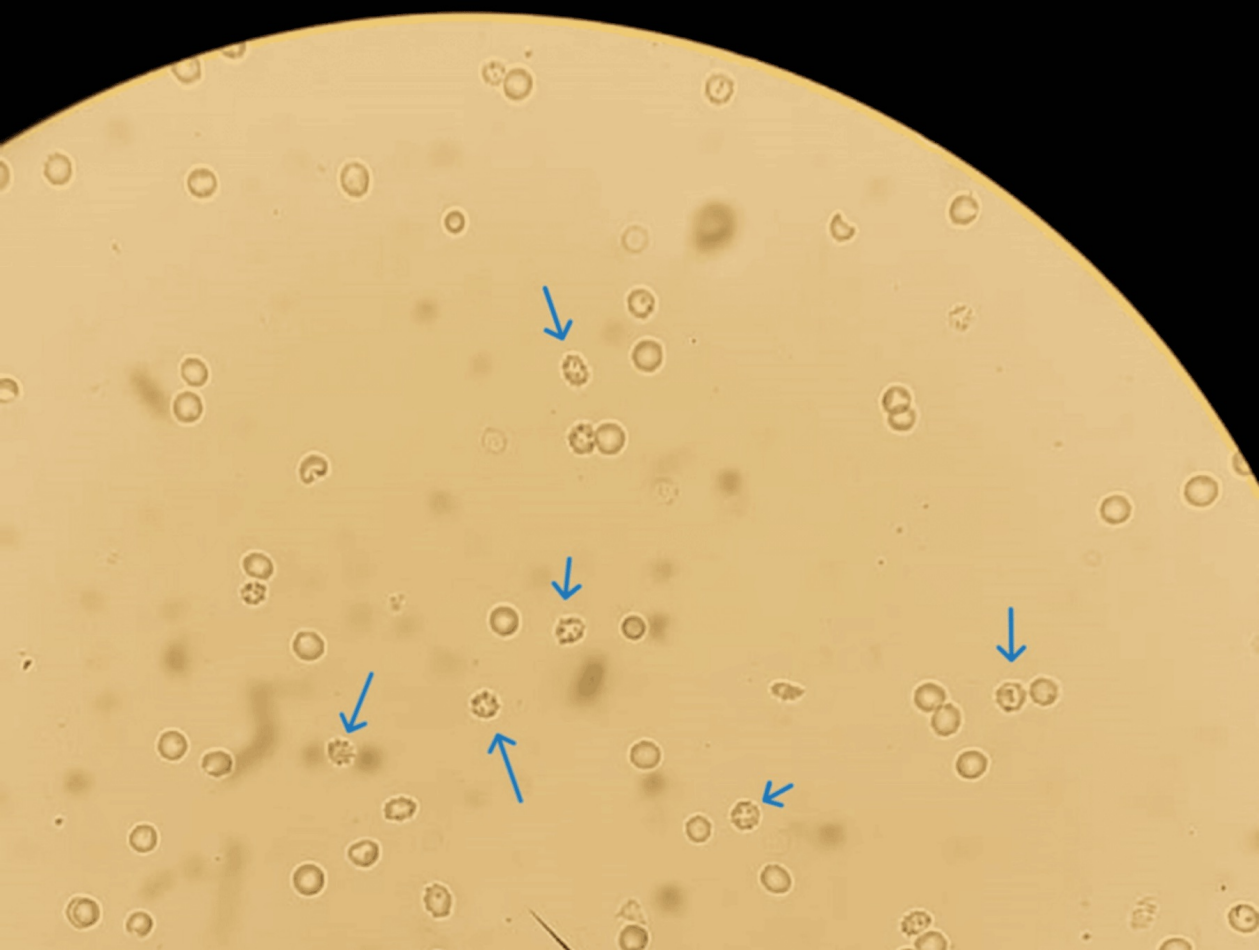
Microscopic urine examination - crenulated red blood cells (blue arrows)

The patient's elevated IgG level (1,650.6 mg/dL) suggests an enhanced immune response. Cardiac enzyme levels were within the normal range: creatine kinase (CK-40 UI/L) and creatine kinase-myocardial band (CK-MB-10 UI/L). Urinalysis revealed the presence of red blood cell casts (50 erythrocytes/uL), with 80% of red blood cells displaying normal morphology and 20% crenulation. The findings, along with a urine protein/creatinine ratio of 7.8 mg/mg, indicate significant glomerular damage. The urine culture was found to be sterile (Figure 1).

**Figure 2:**
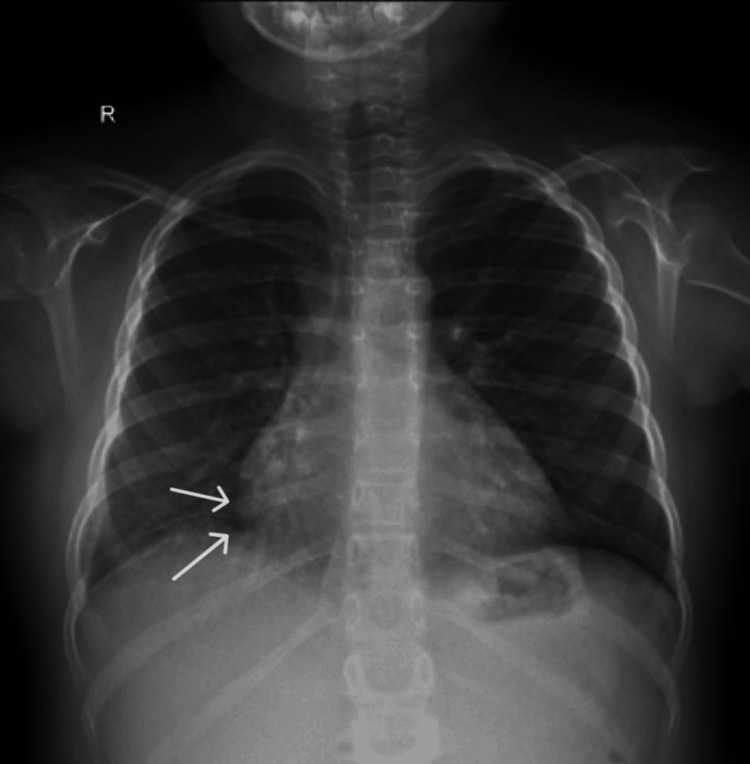
Chest X-ray: Unsystematized alveolar infiltrates ( white arrows) are present in the lower two-thirds of the lung fields, exhibiting a bilateral, peripheral, band-like distribution

A chest X-ray was performed due to the patient's persistent cough, showing unsystematized alveolar infiltrates in the lower two-thirds of the lung fields, with a bilateral, peripheral, band-like distribution. A mixed pattern of diffuse lung infiltrates was observed, which includes nodular alveolar infiltrates and peri-broncho-vascular infiltrates (Figure 2).

**Figure 3:**
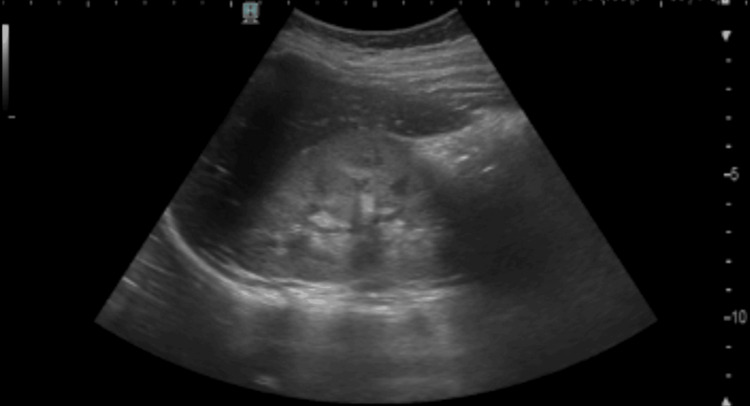
Kidney ultrasound (right kidney)

Furthermore, an abdominal ultrasound showed kidneys exhibiting reduced corticomedullary differentiation, although their size and location were within normal limits. Intraperitoneal fluid measuring 13 mm was noted (Figure 3).

The clinical presentation of the patient suggested systemic vasculitis, particularly granulomatosis with polyangiitis, characterized by constitutional symptoms, including fever, myalgia, and arthralgia, along with multiple organ involvement: otorhinolaryngological manifestations (nasal crusting), pulmonary signs (hemoptysis, pulmonary nodules), and kidney involvement (glomerulonephritis pattern).

**Figure 4:**
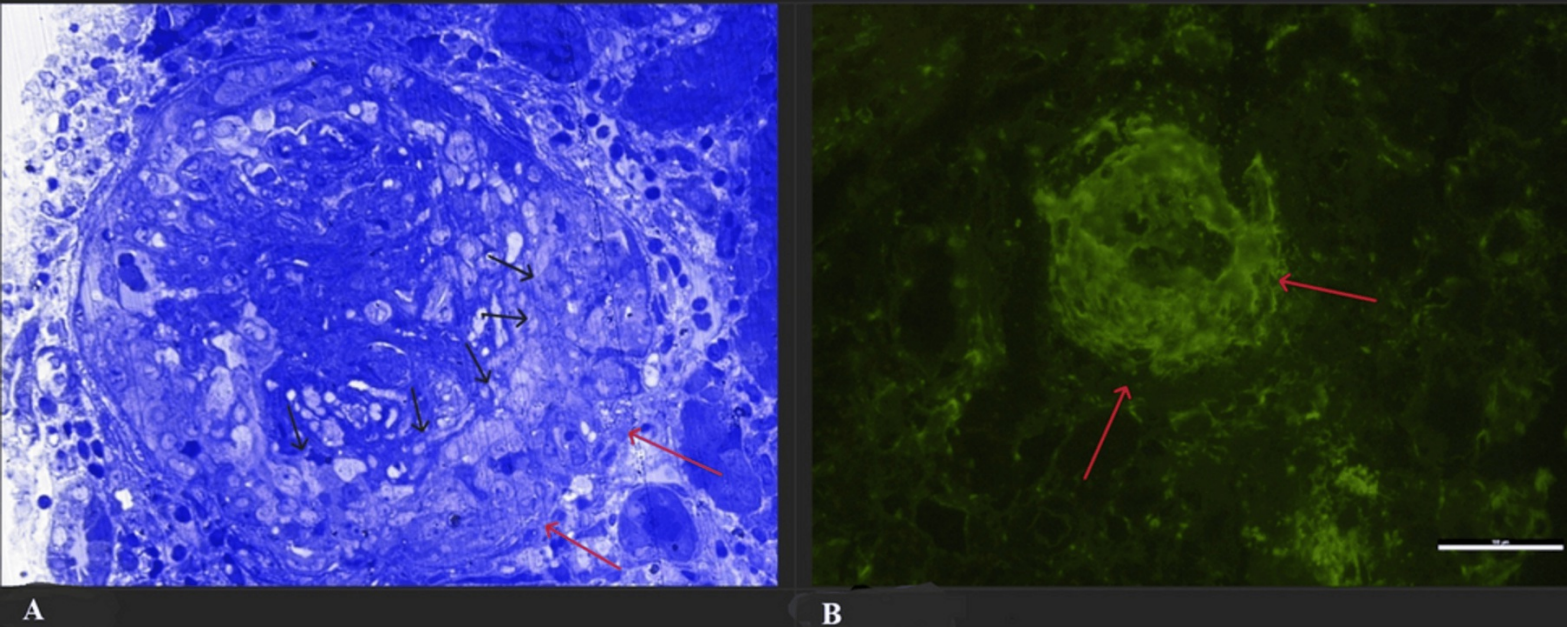
Kidney biopsy (A) Light microscopy revealed cellular crescents (black arrows) in all sampled 20 glomeruli with fibrinoid necrosis and Bowman's capsule destruction (red arrows). (B) Immunofluorescence presents linear glomerular capillary wall positivity, circumferential IgG deposits, and cellular crescents (red arrows).

A kidney biopsy was conducted two weeks after admission. The results, available three weeks later, confirmed pauci-immune glomerulonephritis characterized by fibrinoid necrosis and cellular crescents involving more than 90% of the glomeruli (Figure 4).

**Figure 5:**
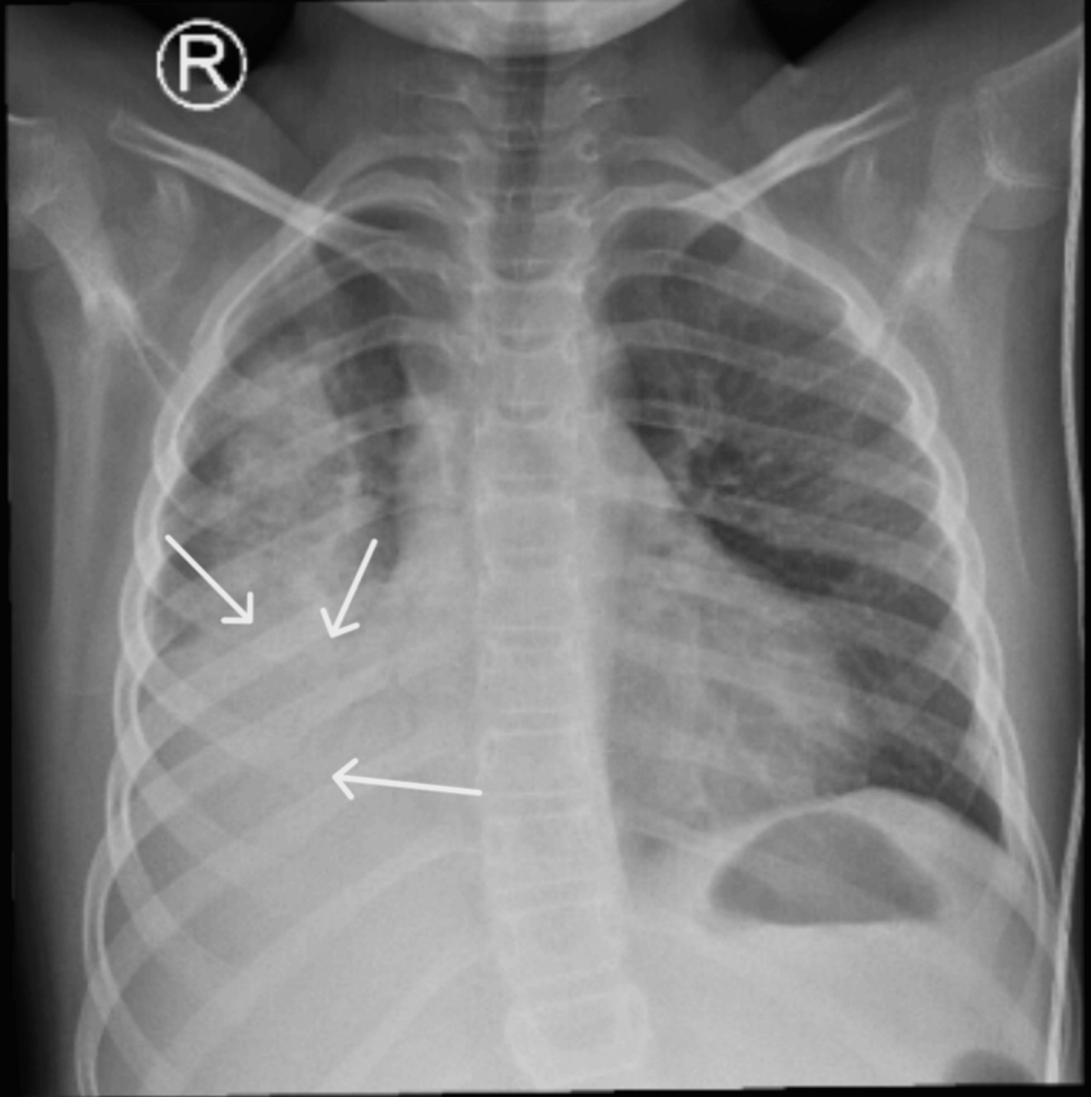
Chest X-ray: consolidation in the right lower and middle lobes (white arrows) indicative of lobar pneumonia

During evolution, nitrogen retention was maintained, and the patient exhibited episodes of hematemesis; recurrent epistaxis, which resolved spontaneously; and a positive occult blood test in the stools. Methylprednisolone pulse therapy was administered for three consecutive days, subsequently transitioning to oral prednisone at a dosage of 1 mg/kg daily. The patient received an initial dose of 500 mg/m^2^ of CYC in addition to remission induction therapy. Following this dose, she developed pneumonia in the right lower and middle lobes, accompanied by hemoptysis (Figure 5).

Plethysmography revealed mild restrictive ventilatory dysfunction characterized by a 24% reduction in vital capacity and a significantly decreased gas transfer factor across the alveolar-capillary membrane.

Throughout her treatment, the patient experienced an episode of desaturation to 88%, which was corrected with oxygen therapy delivered via mask at a rate of 6 L/min, along with inhalation therapy. The treatment regimen included nebulized tranexamic acid and intravenous antibiotics.

Following the administration of the second dose of CYC, the patient experienced a generalized seizure, accompanied by transient aphasia and confusion, necessitating the commencement of anticonvulsant therapy with levetiracetam.

**Figure 6:**
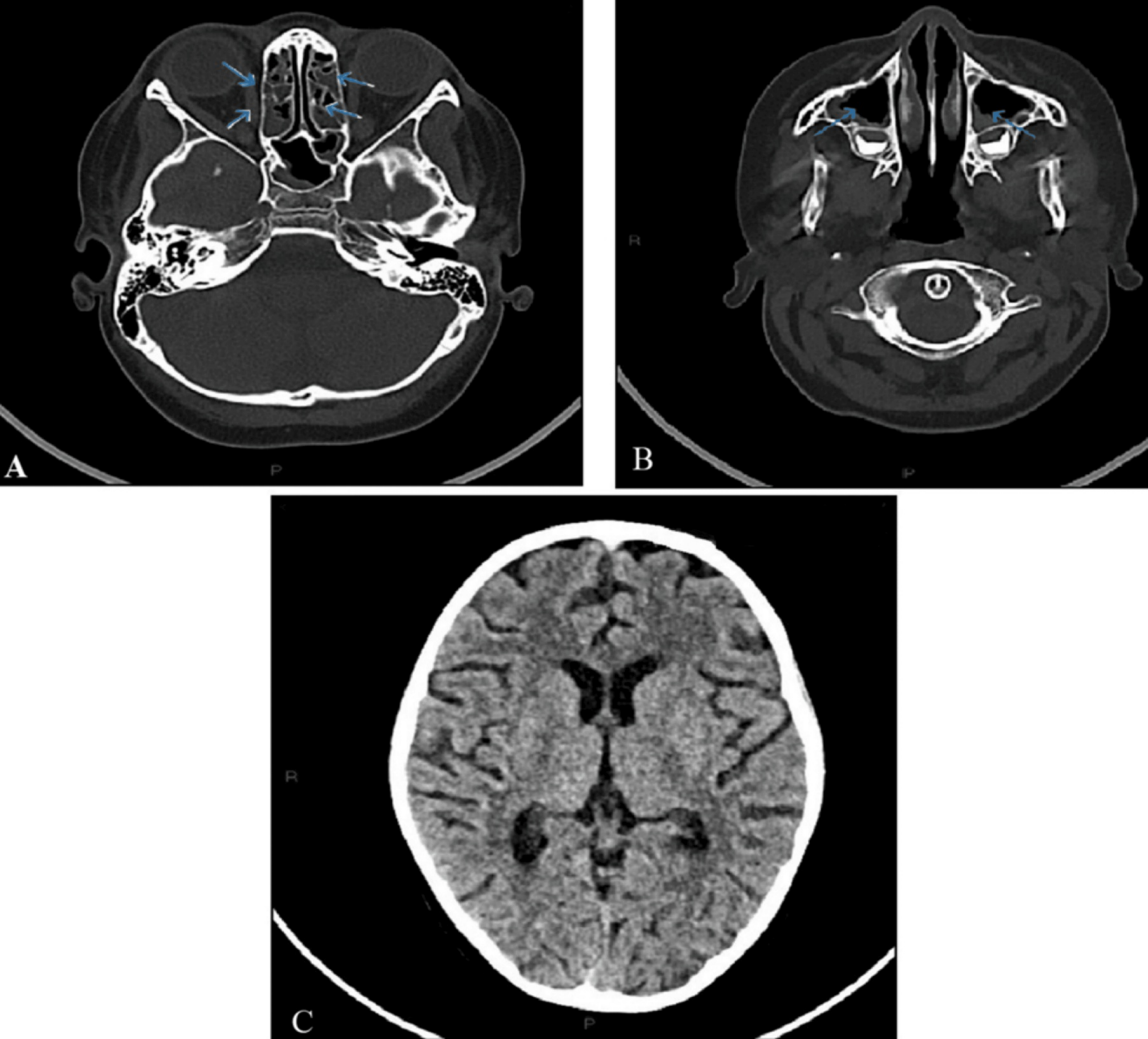
Cerebral CT (A) Ethmoidal sinusitis (blue arrows), (B) maxillar sinusitis (blue arrows), and (C) cerebral atrophy

A lumbar puncture was performed, indicating an elevated chloride concentration, whereas proteinuria and glucosuria levels were within normal ranges. Cerebral CT revealed cerebral atrophy and maxillo-ethmoidal sinusitis, with no indications of acute cerebral changes (Figure 6).

**Figure 7:**
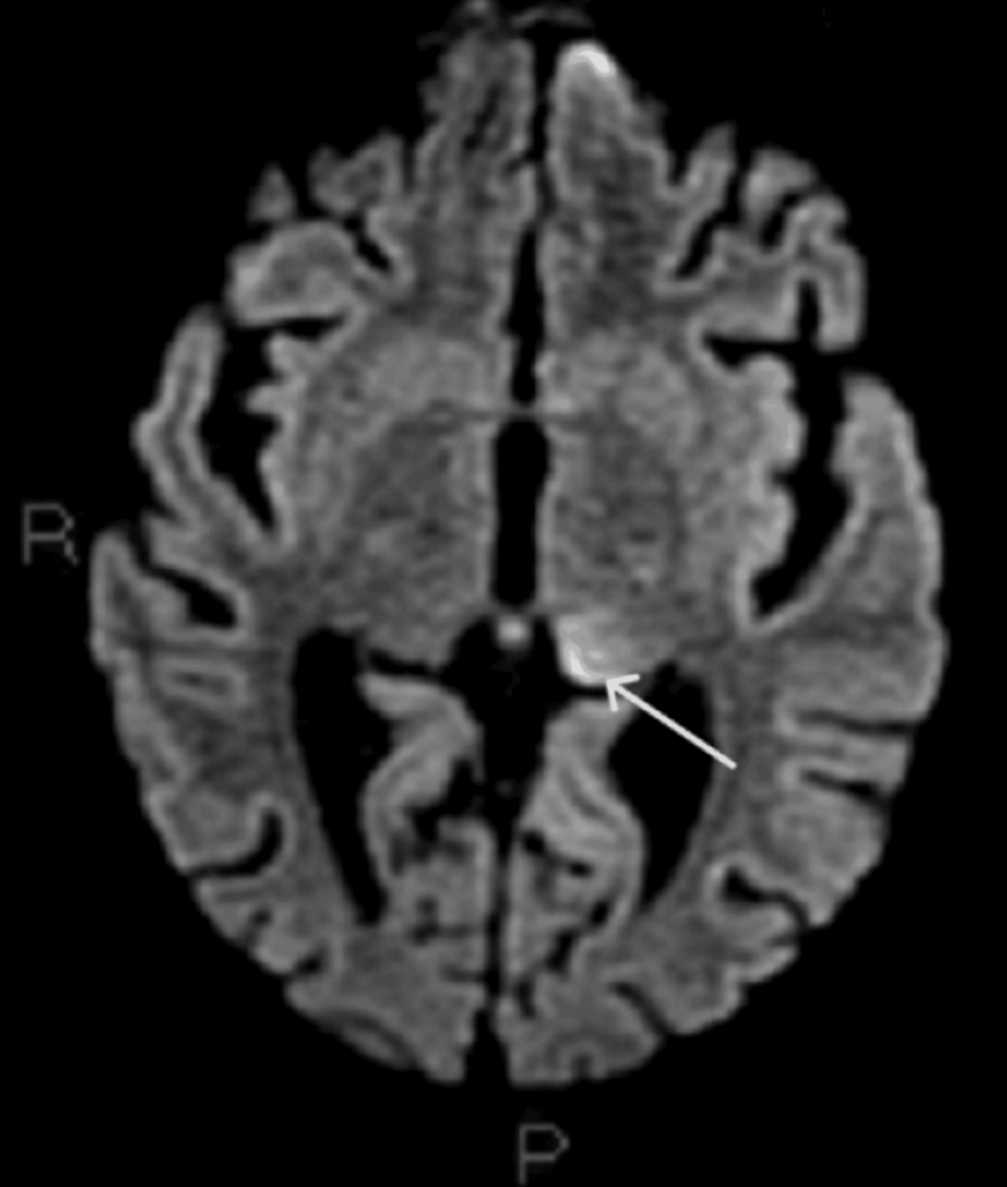
Cerebral MRI Left thalamic signal abnormality (white arrow)

The cerebral MRI indicated a left thalamic signal abnormality, implying a possible subacute ischemic event (Figure 7).

Given the inadequate tolerance to CYC, we decided to switch to RTX at a dosage of 375 mg/m^2^, delivered in four doses at one-week intervals, accompanied by prophylactic treatment with sulfamethoxazole and trimethoprim. Prior to the commencement of rituximab therapy, immunophenotyping was performed, revealing CD19 B lymphocyte levels within the normal range.

After the administration of the second dose of RTX, a minor elevation in inflammatory markers was observed. The clinical examination indicated a decrease in bilateral vesicular breath sounds. Chest X-ray findings revealed alveolar infiltrates, leading to the commencement of a 10-day course of antibiotics (ceftriaxone).

Subsequent to the administration of the fourth dose of RTX, a decline in the patient's condition was observed, characterized by nausea and moderate anterior epistaxis. In the following period, she had three febrile seizures, with temperatures reaching approximately 38.3 degrees Celsius. Blood cultures yielded negative results, leading to an adjustment in treatment, which included intravenous ceftazidime for a duration of seven days and tranexamic acid aerosols for five days.

The patient demonstrated a swift decline in kidney function, as evidenced by an eGFR (calculated with bedside Schwartz formula) reduction exceeding 50% within a three-month period (from 40.67 mL/min/1.73 m^2^ to 10.38 mL/min/1.73 m^2^). The decline was associated with oliguria, hydroelectrolytic disorders, a 3 kg increase in total body weight over six days, and hypertension (maximum value 150/110 mmHg despite treatment with two antihypertensive medications). Hemodialysis for kidney replacement therapy was commenced (Figure 4).

One-month post-final RTX administration, CD19 B lymphocyte levels decreased to 0, requiring a single dose of intravenous immunoglobulin at 0.5 g/kg. In addition, intravenous pulse therapy with methylprednisolone (three doses of 650 mg each) was administered, while discussions regarding the potential readministration of CYC were ongoing.

During treatment, the patient demonstrated variable blood pressure, with maximum readings of 166/100 mmHg, alongside several paroxysmal episodes. The episodes were characterized by confusion, blurred vision, and generalized tonic-clonic seizures accompanied by rightward eye deviation, followed by transient aphasia.

A cerebral CT scan revealed no acute intracerebral lesions; however, there was slight progression of supratentorial chronic ischemic lesions, likely attributable to degenerative microangiopathy, compared to the prior examination. Cerebral and cerebellar atrophy remained relatively stable.

The neurological consultation indicated a clinical and radiologic suspicion of a transient ischemic attack (TIA). In accordance with the neurologist's recommendations, treatment commenced with clexane (0.4 mL subcutaneously once daily) and levetiracetam at a dosage of 1,000 mg per day. The patient displayed visual disturbances, including an inverted perception of objects, fear of cables, and the perception of her father with an infantile look. Furthermore, she exhibited an instance of expressive aphasia, articulating, ‘I can’t say what color it is, although I know’.

**Figure 8:**
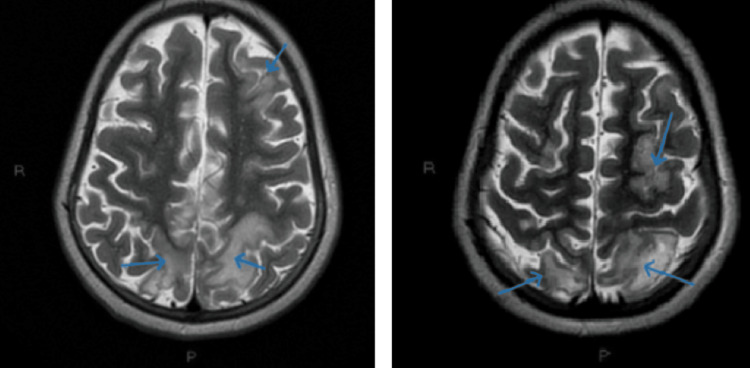
Cerebral MRI - hyperintensities (blue arrows) specific for PRES

**Figure 9:**
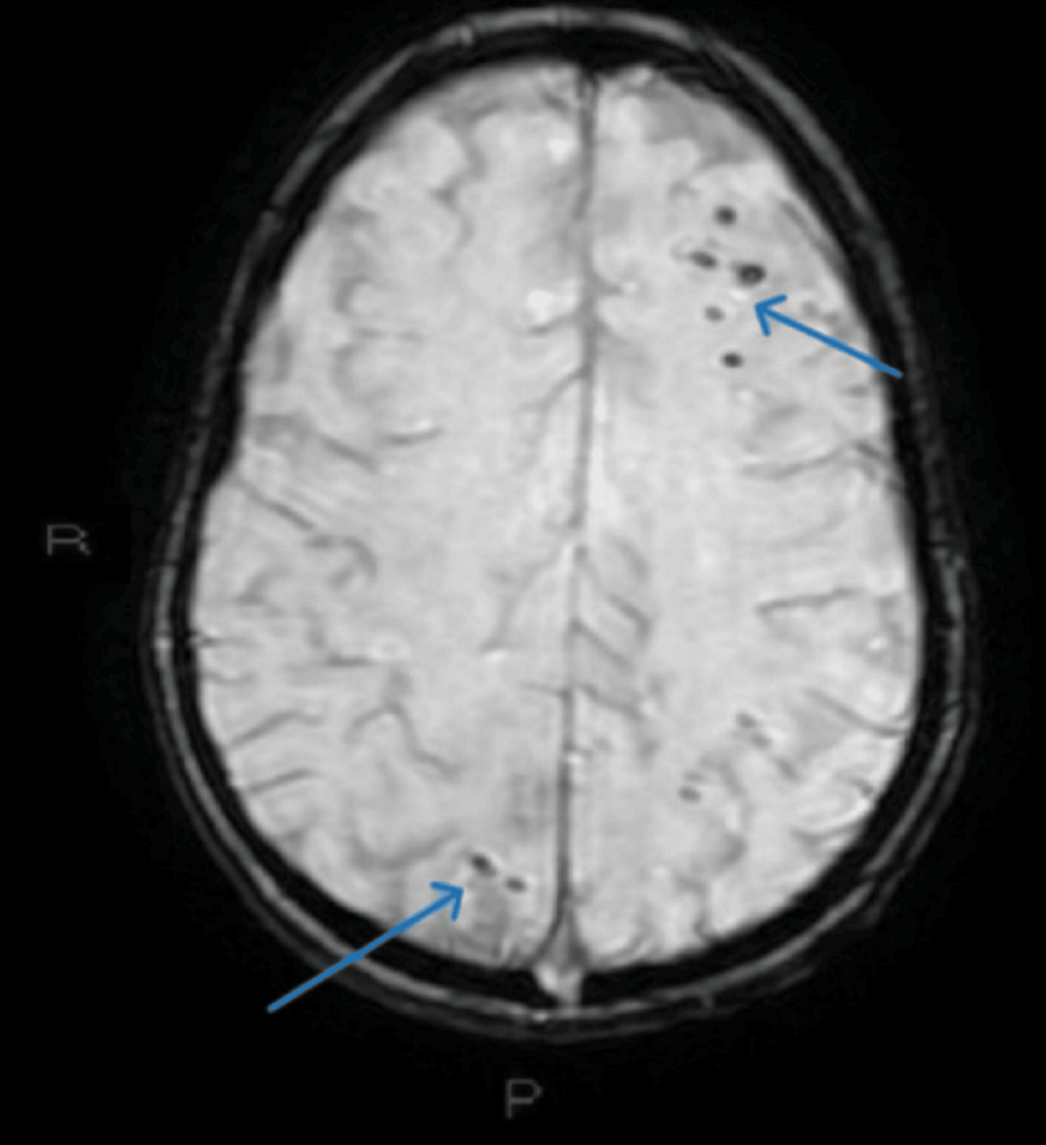
Cerebral MRI - multiple thrombi in the cerebellar and cortical regions (blue arrows)

MRI findings indicated the presence of PRES along with multiple thrombi in the cerebellar and cortical regions (Figures 8-9).

Anticoagulation therapy with enoxaparin was initiated at a prophylactic dose, subsequently transitioning to a therapeutic dose of 3,000 UI after 12 hours. This treatment resulted in signs of hemorrhage, such as bleeding at the catheter and venous puncture sites, and a rapid decline in hemoglobin levels, requiring the administration of red blood cell mass. As a result, anticoagulation therapy was terminated. Extended coagulation tests, thrombophilia profile, and phospholipid syndrome testing were conducted, identifying a homozygous C667T mutation in the MTHFR gene along with elevated homocysteine levels.

The patient's condition advanced with ongoing hypertension, unresponsive to complex oral antihypertensive therapy. Intravenous nicardipine infusion was initiated at a rate of 1.8 micrograms/kg/minute, leading to significant edema in the cephalic region. CT angiography ruled out inferior vena cava syndrome and identified moderate bilateral pleural effusions, significant pericardial effusion, and bilateral pulmonary ventilation abnormalities. Managing blood pressure was challenging, necessitating the administration of various antihypertensive medications, both intravenous and oral.

During hospitalization, the patient exhibited mood disturbances and sleep disorders, including nightmares, which necessitated a consultation with pediatric psychiatry. The patient received a diagnosis of moderate depression and was treated with Prozac (fluoxetine) in conjunction with a tailored psychotherapeutic intervention. After four days of Prozac, the patient exhibits significant drowsiness and bradylalia, likely resulting from cumulative side effects in conjunction with antihypertensive medication, alongside an improvement in the patient's condition, including mood disorders. Prozac was discontinued, leading to an improvement in mood and cognitive symptoms. However, ongoing psychotherapeutic support was deemed necessary due to the chronic nature of her condition, extended hospitalization, and the complexity of her treatment, which involved adaptation to a dietary regimen, hemodialysis, and multiple antihypertensive medications.

## Discussion

**Figure 10:**
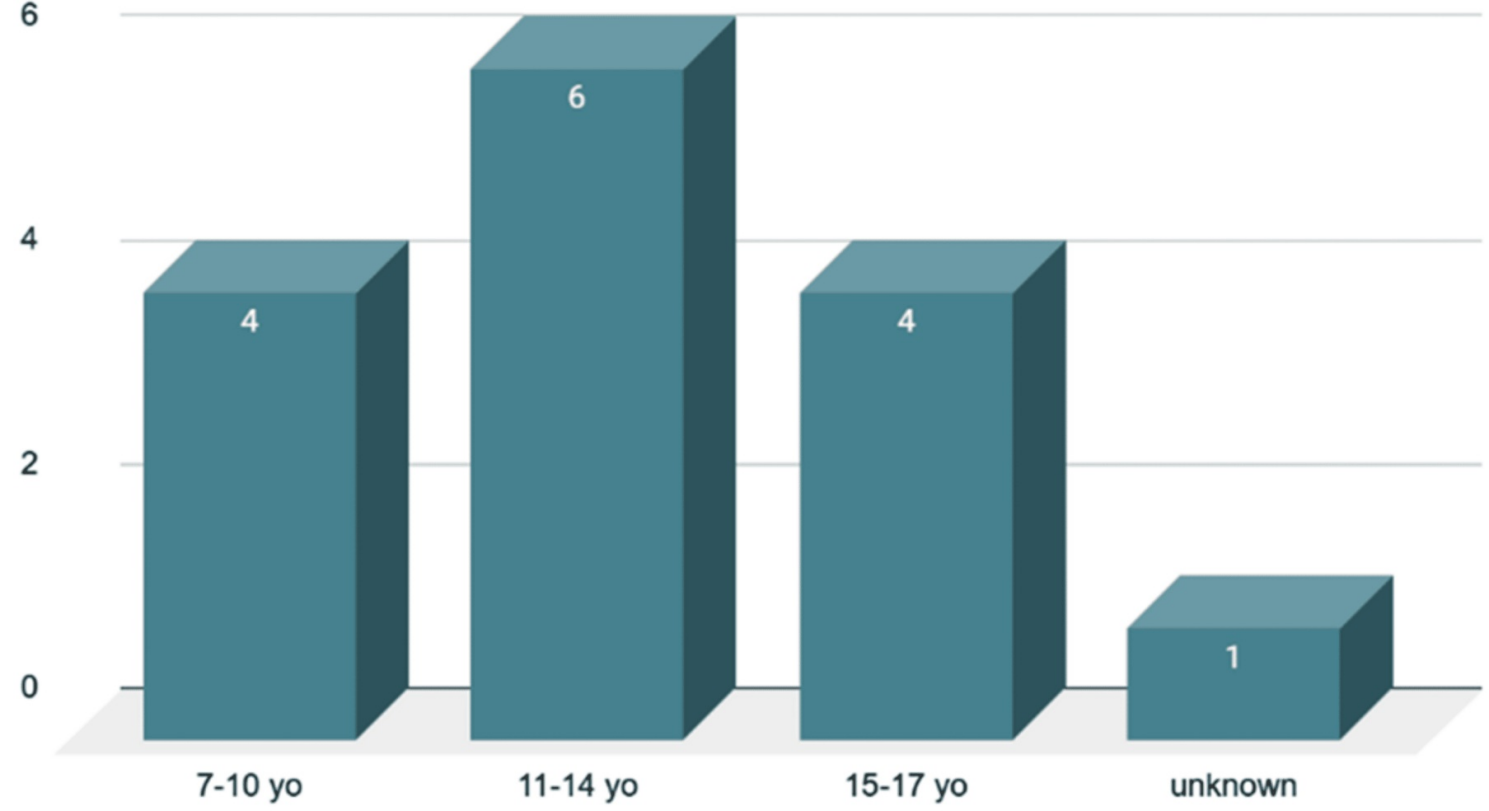
Age of the onset in the review study

**Figure 11:**
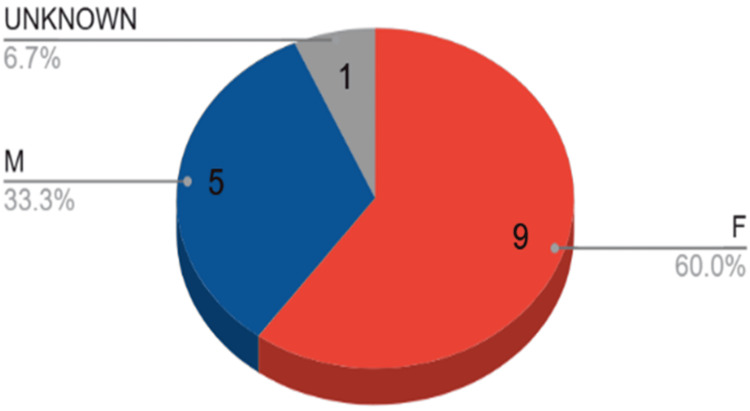
Sex predominance in the review study M - male; F - female

The age of onset represents a significant distinction between the case report and the cohort. The patient, at seven years old, is below the cohort's average age of 12, which spans from seven to 17 years. In the reviewed cases, only one individual was as young as the youngest participant, two others were under 10 years old, while the majority were older. This indicates that, while GPA may occur in very young patients, it is more frequently diagnosed in older children and adolescents. Furthermore, the cohort was primarily female, aligning with trends identified in GPA epidemiology; however, this case offers an additional understanding of gender dynamics in early-onset presentations (Figures 10-11) [3-15].

In the reviewed cases, the respiratory system was the most affected, with eight out of 15 patients presenting respiratory symptoms upon admission [5,7,8,12-15]. The girl presented notable symptoms, such as cough and hemoptysis, while other cases reported analogous manifestations, particularly cough, asthma, and dyspnea. Common symptoms, including fever, asthenia, and weight loss, were reported with similar frequency, closely matching the clinical presentation noted in our case [1,3-5,7,12-15]. Additionally, both the individual case and the cohort displayed significant ENT symptoms, such as recurrent epistaxis and chronic rhinitis [1,5,7,13,15]. In the examined cohort of patients, seven exhibited arthralgia in multiple joints [3,5,7,9,10,12,15], while three experienced myalgia at onset [7,10,12]; these symptoms were included in patients' medical history upon admission to the hospital. Skin lesions, such as purpuric lesions and ulcerations, were moderately common in the cohort, affecting four patients, and were significantly more severe than the girl's presentation [1,9,10,15]. Nausea and vomiting were less commonly reported in the reviewed cases, with only two instances noted, whereas our case included these symptoms at admission [3,11]. Furthermore, three patients in the cohort presented with ophthalmologic symptoms [4,6,9], while another three experienced cardiovascular issues, which were not observed in our case [11,12,14]. Headaches were observed in two of the published cases and in our case, suggesting potential central nervous system involvement and the associated risk of complications [1,10].

**Figure 12:**
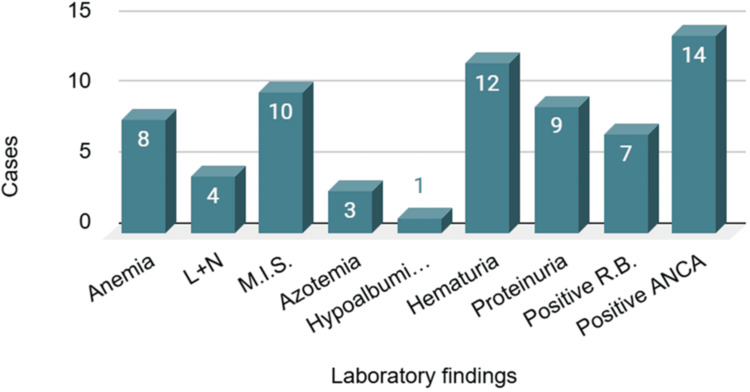
Laboratory investigations in the review study L+N - leukocytosis and neutrophilia; M.I. S - marked inflammatory syndrome; R.B. - renal biopsy

Laboratory results indicate both similarities and differences. Hematuria and proteinuria were the predominant findings in the cohort, consistent with the renal complications observed in our case report. Anemia, neutrophilia, and a pronounced inflammatory syndrome were observed in many cases, suggesting a systemic inflammatory response, similar to that of our patient. In our study, elevated levels of urea, creatinine, and uric acid were observed; however, these abnormalities were present in only three out of the fifteen patients examined. Hypoalbuminemia was observed in only one patient from the analyzed cohort. Antineutrophil cytoplasmic antibodies (p-ANCA or c-ANCA) were positive in 14 of the 15 reviewed cases, including our case. The positive renal biopsy in this case reinforces the diagnosis and aligns with findings from the cohort, which included renal biopsies in seven cases and positive tissue biopsies in an additional seven patients, thereby confirming GPA (Figure 12).

**Figure 13:**
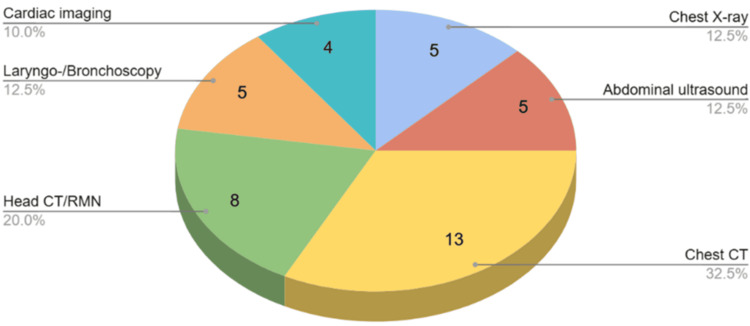
Imaging investigations in the review study

Multiple investigations were performed within the cohort to evaluate the degree of organ involvement and the progression of the disease. Chest CT was conducted in 13 of the 15 patients, with all scans demonstrating diffuse bilateral cavitary lesions. An MRI/CT examination of eight patients revealed cystic masses, intraorbital lesions, and subglottic stenosis, but it was sinus-related abnormalities, particularly sinusitis, that were most frequently detected. Chest X-rays were performed on five patients, revealing cavitary opacities in each instance. Abdominal and renal ultrasound was performed on five patients, indicating enhanced renal cortex and increased kidney size, which suggests renal involvement. Cardiac investigations, including echocardiography and Doppler studies, were conducted in four patients, revealing pericardial effusion. Laryngoscopy and bronchoscopy were conducted in five patients, revealing notable airway obstruction and lesions. Findings included multiple firm lesions in the trachea, severe subglottic stenosis, diffuse airway bleeding, and friable mucosa (Figure 13).

Our study demonstrated a consistent requirement for pulse therapy with methylprednisolone in the treatment of GPA across all cases. Ten patients maintained oral prednisone therapy, while five received CYC and three were treated with azathioprine, based on their individual responses and disease severity. RTX was administered to eight patients as part of their treatment regimen. Most patients required broad-spectrum antibiotic therapy and antifungal treatment to manage infections frequently associated with GPA. Symptomatic management and treatment of complications were essential, involving a variety of interventions, including ocular prednisolone eye drops, intravenous immunoglobulins, antihypertensive medications, anticoagulants, and other supportive therapies customized to meet individual patient requirements. In the analysis of our case, it is essential to observe that the patient underwent treatment in accordance with the established protocol, which comprised pulse therapy with methylprednisolone, succeeded by oral prednisone at a dosage of 1 mg/kg/day, CYC, and, subsequently, RTX. Most patients in the cohort followed this methodology. The patient encountered numerous complications, each necessitating specific treatments. The management of infections required extended and intensive antibiotic therapy, a significant element of the overall treatment plan. The patient required medication for complications, including diuretics, antihypertensives, and various forms of supportive care.

Complications

The patient encountered multiple complications, notably severe infectious conditions, including right lower and middle lobe pneumonia and hemoptysis, which manifested at admission and following the initial dose of CYC. Renal complications encompassed rapidly progressive glomerulonephritis accompanied by nephrotic syndrome, hypertension, and end-stage kidney disease necessitating hemodialysis. The patient exhibited generalized seizures, transient aphasia, and confusion after the second dose of CYC. Imaging indicated subacute ischemic lesions, which subsequently resolved completely. Additional neurological complications comprised tonic-clonic seizures and visual disturbances. Moreover, the patient experienced notable hemorrhagic events, pronounced hyponatremia, and psychiatric complications, including panic attacks and depression. The cohort of children displayed a diverse array of complications, with some resembling those seen in our patient and others being unique. Pulmonary complications were prevalent in the cohort, with four children suffering from severe infections, pulmonary embolism, alveolar hemorrhage, and extensive sinopulmonary inflammation, resulting in respiratory failure and necessitating mechanical ventilation in two instances. Cardiovascular complications were significant, with three patients experiencing conditions, including cardiac tamponade, elevated troponin-T and BNP levels, and pericardial effusions. The conditions underscore the variability in cardiovascular involvement within the cohort. One patient exhibited PRES, a complication also observed in our case, underscoring the complications associated with severe vasculitis and the additional stressors affecting the brain. Additionally, one child exhibited notable ocular involvement characterized by keratitis and episcleritis, while two others presented with sinus and orbital complications, including orbital pseudotumor. In the cohort, ENT complications included airway involvement, with two patients experiencing severe tracheal stenosis and subglottic stenosis, complicating their management.

Evolution

Clinical outcomes varied among the cohort of patients. Multiple patients demonstrated initial improvement, including the resolution of skin lesions and the normalization of renal function. Significant relapses were addressed using treatments such as oral prednisone, methotrexate, and plasmapheresis. One patient exhibited recurrent PR3-ANCA elevation and proteinuria following the tapering of prednisone, but stabilization was achieved with methotrexate and an increased dosage of prednisone. A different patient exhibited notable improvement in lung cavitations and was subsequently treated with CYC following the exacerbation of hemoptysis. Certain patients underwent surgical interventions, including palatal defect closure and orbital pseudotumor surgery, resulting in favorable outcomes. Several patients experienced complications such as pulmonary thromboembolism, severe anemia, and respiratory distress, necessitating ICU admission and intensive therapies, including plasmapheresis, CYC, and mechanical ventilation. Following several months of treatment, most patients attained remission, with continuous monitoring indicating stable disease, normal inflammatory markers, and decreased ANCA levels. Following discharge from our clinic, the patient was admitted to The Clinical Institute of Urology and Kidney Transplant in Cluj, where she underwent a kidney transplant in February 2024. She is currently receiving treatment with Mycophenolate Mofetil, demonstrating positive progress.

## Conclusions

This case is notably more complex and severe than a cohort of 15 children, due to the combination of rare and interrelated medical issues and an early onset. The seven-year-old patient exhibited significant hypertension and acute renal failure, requiring hemodialysis intervention. Additionally, she developed PRES, which further complicated her clinical presentation. Genetic analysis identified a homozygous C667T mutation in the MTHFR gene, which is associated with hyperhomocysteinemia and folic acid deficiency. She suffered a rapid and severe progression of her illness as a result of a combination of these factors, along with numerous cortical and cerebellar microthrombi. The reviewed cases presented several severe complications; however, the combination of genetic, vascular, and neurological issues in this instance renders it significantly more complex and challenging compared to the cohort. This case demonstrates a more rapid progression and multiple life-threatening complications necessitating urgent and specialized management.

This study aims to better understand the complexities and challenges involved in the diagnosis and management of early-onset GPA in pediatric patients. The differences in age, gender distribution, and clinical presentation highlight the necessity for heightened awareness, especially in younger patients where GPA may manifest in an unusual manner. The individual case and the cohort exhibited numerous clinical and laboratory findings, especially in respiratory, renal, and systemic symptoms, highlighting shared manifestations and complications, including severe infections, renal impairment, and neurological involvement. Imaging and biopsy findings were crucial in confirming the GPA diagnosis. The treatment regimens, primarily involving corticosteroids, CYC, and RTX, were essential for managing the systemic effects of the disease and achieving remission. This case highlights the significance of personalized and multifaceted treatment strategies to manage the varied and severe complications linked to GPA, which necessitate comprehensive supportive care. Despite the high risk of morbidity and mortality associated with GPA without timely intervention, the sustained and tailored therapeutic approaches noted in these cases indicate considerable potential for disease stabilization and enhanced long-term outcomes in pediatric patients.

## References

[1] Knopp BW, Baran J, Casey R (2023). Childhood-onset granulomatosis with polyangiitis as a palatal defect: a case report. Cureus.

[2] Filocamo G, Torreggiani S, Agostoni C, Esposito S (2017). Lung involvement in childhood onset granulomatosis with polyangiitis. Pediatr Rheumatol Online J.

[3] Saenz Rios F, Devaraj S, Movva G, Movva H, Nguyen QD (2020). Granulomatosis with polyangiitis in a pediatric male. Cureus.

[4] Kato M, Jimbo K, Nagata M (2021). Novel pediatric granulomatosis with polyangiitis with a marked bloody pericardial effusion and bloody stool: a case report. Allergy Asthma Clin Immunol.

[5] Shelton A, Parikh S, Mims C, Quintero-Del-Rio A (2023). A challenging case of granulomatosis with polyangiitis with cardiac involvement: a rare case report. AME Case Rep.

[6] Ure E, Kayadibi Y, Sanli DT, Hasiloglu ZI (2016). Orbital involvement as the initial presentation of Wegener granulomatosis in a 9-year-old girl: MR imaging findings. Diagn Interv Imaging.

[7] Jari M (2021). Thrombosis as the first manifestation of granulomatosis with polyangiitis disease in an adolescent. Case Rep Hematol.

[8] Lee PY, Adil EA, Irace AL (2017). The presentation and management of granulomatosis with polyangiitis (Wegener's granulomatosis) in the pediatric airway. Laryngoscope.

[9] Amini S, Jari M (2023). Granulomatosis with polyangiitis misdiagnosed as IgA vasculitis in a child. Case Rep Pediatr.

[10] Javadi Parvaneh V, Shirzani A, Rahmani K, Shiari R (2020). Pediatric granulomatosis with polyangiitis mimicking IgA vasculitis: a case report. Clin Med Insights Arthritis Musculoskelet Disord.

[11] Kuang Q, He X, Jia L, Zhang Z, Gui C, Gao C, Xia Z (2023). Case report: a pediatric case of MPO-ANCA-associated granulomatosis with polyangiitis superimposed on post-streptococcal acute glomerulonephritis. Front Pediatr.

[12] Figueiredo R, Pires Duro I, Marinho A, Mota C, Guedes M, Zilhão C (2021). Granulomatosis with polyangiitis in adolescence: two distinct presentations. Case Rep Rheumatol.

[13] Konstantinidou S, Coyle P, Pandey G, Butler C (2021). Multilevel tracheal granulomatosis with polyangiitis (GPA) lesions in a paediatric patient. BMJ Case Rep.

[14] Azin N, Hajihashemi A, Geravandi M (2023). Rare central nervous system manifestation of granulomatosis with polyangiitis in a 12-year-old child: a case report. Radiol Case Rep.

[15] Pathania S, Rehman R, Ward M, Yalcindag A, Ross A, Herzlinger M, Gorbounova I (2024). An unusual case of pediatric granulomatosis with polyangiitis complicated by splenic infarction presenting as inflammatory bowel disease. JPGN Rep.

